# AUGUSTUS at EGASP: using EST, protein and genomic alignments for improved gene prediction in the human genome

**DOI:** 10.1186/gb-2006-7-s1-s11

**Published:** 2006-08-07

**Authors:** Mario Stanke, Ana Tzvetkova, Burkhard Morgenstern

**Affiliations:** 1 Institut für Mikrobiologie und Genetik, Universität Göttingen, Goldschmidtstraße, 37077 Göttingen, Germany

## Abstract

**Background:**

A large number of gene prediction programs for the human genome exist. These annotation tools use a variety of methods and data sources. In the recent ENCODE genome annotation assessment project (EGASP), some of the most commonly used and recently developed gene-prediction programs were systematically evaluated and compared on test data from the human genome. AUGUSTUS was among the tools that were tested in this project.

**Results:**

AUGUSTUS can be used as an *ab initio *program, that is, as a program that uses only one single genomic sequence as input information. In addition, it is able to combine information from the genomic sequence under study with external hints from various sources of information. For EGASP, we used genomic sequence alignments as well as alignments to expressed sequence tags (ESTs) and protein sequences as additional sources of information. Within the category of *ab initio *programs AUGUSTUS predicted significantly more genes correctly than any other *ab initio *program. At the same time it predicted the smallest number of false positive genes and the smallest number of false positive exons among all *ab initio *programs. The accuracy of AUGUSTUS could be further improved when additional extrinsic data, such as alignments to EST, protein and/or genomic sequences, was taken into account.

**Conclusion:**

AUGUSTUS turned out to be the most accurate *ab initio *gene finder among the tested tools. Moreover it is very flexible because it can take information from several sources simultaneously into consideration.

## Background

With an increasing number of completely or partially sequenced genomes, computational prediction of protein-coding genes has become one of the most active fields of research in bioinformatics. This task is particularly challenging for eukaryotes, where protein-coding exons are usually separated by non-coding introns of varying length. Previous studies have shown that the accuracy of the currently available tools for gene finding in human is not satisfactory [1].

AUGUSTUS is a method for gene finding in eukaryotes [2]. The original version of the program used intrinsic information only, that is, information contained in the genomic sequence that is to be annotated. A recent extension of the program is also able to integrate extrinsic information from arbitrary sources for improved prediction accuracy [3].

At the ENCODE genome annotation assessment project (EGASP) workshop that took place in May 2005 in Cambridge, UK, some of the currently used methods for gene prediction were systematically evaluated and compared, including some of the most widely used gene-finding tools [4,5]. AUGUSTUS was among the methods that were evaluated at this workshop.

## Results and discussion

*Ab initio *gene prediction is an important tool for the task of finding new genes for which sufficient evidence from transcribed sequences is not available. It is particularly important in genome projects of species where a large fraction of the genes cannot be constructed using expressed sequence tag (EST) evidence. *Ab initio *gene prediction is typically one of the first annotation steps in eukaryotic genome projects. For the test set of EGASP, the predictions of five *ab initio *single genome programs were evaluated by the organizers of the workshop [6]. Besides AUGUSTUS, these programs were GENSCAN [7], GeneID [8], GeneMark.hmm [9] and Genezilla [10]. All programs predicted only the coding parts of the genes and only one transcript per gene. On the gene and transcript level AUGUSTUS outperformed all the other programs with respect to both sensitivity and specificity. AUGUSTUS achieved a gene level sensitivity of 24.3% and a gene level specificity of 17.2%; for about one-quarter of the genes it predicted one splice variant exactly as annotated and 17.2% of the genes predicted by AUGUSTUS are correct according to the annotation. The second most sensitive program, Genezilla, had a gene level sensitivity of only 19.6% and also had the disadvantage that it predicted many more false positive genes. Only 8.8% of the genes predicted by Genezilla were correct. The second most specific program on the gene level after AUGUSTUS was GENSCAN. But even GENSCAN had a gene level specificity of only 10.1% and a gene level sensitivity of only 15.5%.

On the base and exon level the situation was less clear. Taking the mean between sensitivity and specificity, however, AUGUSTUS also had the best values on the base and exon level, very closely followed by GeneID. Apparently, GeneID is as good as AUGUSTUS at finding exons but is less successful at chaining the exons to genes: compared to AUGUSTUS, it correctly predicted less than half the number of genes. Also, the fraction of predicted genes that are correct is about half the number predicted by AUGUSTUS.

In the category of genome-genome comparisons, the predictions of eight programs were evaluated with respect to their ability to predict the coding regions of genes. The program NSCAN [11], which used mouse, rat and chicken as informant genomes, clearly performed best. AUGUSTUS performed second best with respect to the average of sensitivity and specificity at the base level and also with respect to the average of sensitivity and specificity at the exon level. The program MARS, which also uses multiple informant genomes, performed second best at the gene level. According to the average of sensitivity and specificity, AUGUSTUS was the most accurate comparative gene prediction method at the base, exon and gene levels that is based on just one informant species (mouse in the case of AUGUSTUS).

The use of expression data as a source of information improved the accuracy of AUGUSTUS dramatically. For example, the gene level sensitivity increased to 47.6% and the gene level specificity increased to 37%. However, many programs could reconstruct the genes much better than AUGUSTUS.

### What went right?

AUGUSTUS turned out to be the most accurate program among the participating programs and the University of California Santa Cruz (UCSC) hosted programs when no other data than the human genome was used or just one other informative genome was used. Furthermore, the method of incorporating hints makes it a flexible program that can use external information from various sources. The hints are collected by independent programs and stored in a standard file format. AUGUSTUS can use hints from one source alone or use hints from several sources at the same time. This is particularly important for species where one source of hints is not yet available, for example, because a closely related species is not yet sequenced or not enough ESTs are available.

### What went wrong?

Compared to newly sequenced genomes, human genes are, on average, extremely well supported by experimental data. For most of the genes in the EGASP test set there were full length mRNA, ESTs or protein alignments supporting one or more splice variants. When the ESTs, mRNA and protein data are available, the task of gene prediction consists more of reconstructing the (alternative) transcripts from the available evidence than of predicting new genes. This explains why AUGUSTUS compared favorably with the other programs in the absence of extrinsic evidence but was outperformed by some of them when ESTs, mRNA and protein data were available. Our method of finding evidence using EST and protein BLAST alignments is more geared towards weak evidence, for example, evidence from other species. In the presence of a large number of human ESTs and even full length cDNAs, spliced alignment should be preferred over BLAST alignments. This is particularly so because, in contrast to BLAST, spliced alignment methods assume the presence of long gaps corresponding to introns and such methods are likely to be more precise at inferring intron boundaries. Another disadvantage of our program that makes hints from protein alignments is the fact that it treats alignments with human sequences the same as alignments with sequences from other species.

## Conclusion

For genomes with extensive high quality expression data we should generate hints for AUGUSTUS using spliced alignments, such as from BLAT [12], instead of BLAST alignments. Also, we will work to improve comparative gene prediction using multiple species and more sensible methods for extracting information about the location of splice sites from multiple species genomic alignments. Furthermore, we are currently extending the model to the untranslated regions of genes.

## Materials and methods

### Hints to AUGUSTUS from extrinsic evidence

Evidence about the location of exons, introns and biological signals of a given input DNA sequence *s *can be retrieved in various ways, such as by comparing *s *to genomic sequences of other species or by comparing *s *to ESTs or proteins from a database. We refer to this as 'extrinsic' evidence as it is derived from sources other than the sequence *s *itself. In contrast, 'intrinsic' evidence is evidence derived from the sequence *s *itself, such as a long open reading frame or the occurrence of typical splice site patterns.

The model underlying the program AUGUSTUS has been extended to a model that we call AUGUSTUS+. Both the original model and the extended model are implemented in the same program AUGUSTUS. There is only one version of the program but two different models, depending on whether extrinsic evidence is given as input or not. AUGUSTUS+ incorporates certain pieces of extrinsic evidence, which we call 'hints', as input and balances it with the intrinsic evidence to produce a most likely gene structure that takes both the intrinsic and extrinsic evidence into account.

As our method of incorporating hints has been described in [3,13], we here only describe the practical effects of our model, an extension to it that allows the formulation of hints about introns and its application in EGASP 2005.

Each hint is a piece of information of one of the following types: *start*, the position of a translation start site; *stop*, the position of a translation stop; *ass*, the position of an acceptor splice site; *dss*, the position of a donor splice site; *exonpart*, the interval that is part of an exon; or *exon*, the interval that is exactly an exon.

For the first four types, the hint specifies the sequence position of the biological signal and a strand. *Exonpart *and *exon *hints specify a range of sequence positions, a strand and a reading frame. Each hint is also assigned a grade from a small discrete set of grades that may depend on the type of the hint and the sources of available extrinsic information.

The grade makes it possible to distinguish hints with different degrees of reliability. For example, both alignments with ESTs and protein sequences yield *dss *hints. However, it turns out that those *dss *hints we derive from protein alignments coincide on a training set more often with true donor splice sites. Giving all *dss *hints from proteins one grade and all *dss *hints from ESTs another grade allows us to distinguish their reliability. Another typical application would be to map a score of an alignment to a grade of the hint derived from the alignment; for example, by introducing three grades for a low, medium and a large score. The set of grades is an abstract set; grades are not numbers. The parameters measuring the reliability of the hints and its dependency on the grade are estimated on a training set with known annotation.

The model underlying the program AUGUSTUS is a so called generalized hidden Markov model (GHMM). HMMs and GHMMs for gene prediction typically define a probability for each pair (*φ*,*s*) of a sequence *s *and a gene structure *φ*. Here, the term 'gene structure' refers to a parse of the input sequence into exons, introns and intergenic regions. By contrast, the model AUGUSTUS+ defines a probability distribution on the set of all triples (*φ*,*s,h*), where *h *is a set of hints. Such hints can come from arbitrary sources of additional information that are available to the user, for example, alignments to expressed sequences or any kind of expert information. The distribution is such that the marginal distribution of (*φ*,*s*) is the same as in the *ab initio *AUGUSTUS model not incorporating hints. Given a sequence *s *and a set of hints *h*, AUGUSTUS searches the most likely gene structure *φ *given *s *and *h*, that is, a gene structure satisfying:

*φ *= argmax_*φ*_*p*(*φ*|*s*,*h*)

As in standard HMM theory, this is equivalent to searching a gene structure that satisfies:

*φ *= argmax_*φ*_*p*(*φ*,*s*,*h*)

**Figure 1:**
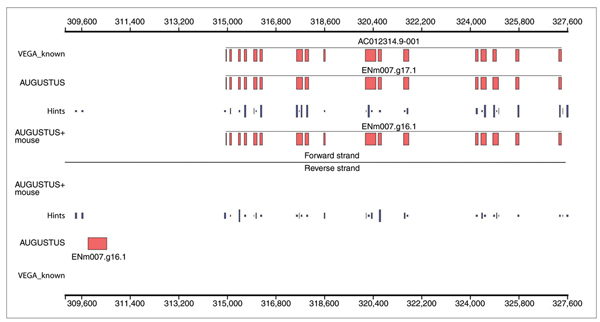
Annotation of the protein coding regions of a part of the human ENCODE region ENm007. The line labeled 'VEGA_Known' shows one known gene on the forward strand. The *ab initio *program AUGUSTUS (labeled 'AUGUSTUS') predicts this gene almost correctly but completely misses the 9th exon annotated around position 318,600. Furthermore, as an *ab initio *program, AUGUSTUS predicts a false positive gene on the reverse strand around position 310,000. The lines labeled 'hints' show the hints derived from a comparison to the mouse genome. The height of the rectangles depends on their estimated reliability. The hints indicate the presence of an exon where AUGUSTUS missed the annotated exon. Also, there are no hints about coding regions where AUGUSTUS predicted a gene on the reverse strand. When the given hints are used by AUGUSTUS (labeled 'AUGUSTUS+mouse'), the missed exon is correctly predicted and the false positive gene is not predicted anymore. The former is a consequence of the bonus effect and the latter a consequence of the malus effect. Note that the hint about the exon around position 318,600 was helpful, although that exon is more likely to be on the reverse strand according to the hints alone. This plot has been obtained using gff2ps [28].

When *s *and *h *are given, we refer to *p*(*φ*,*s*,*h*) as the likelihood of the gene structure *φ*. For the decision which gene structure has the highest likelihood - and is therefore predicted -only the likelihood of the gene structures relative to each other is relevant, not their absolute value. The introducton of hints changes the relative likelihood of gene structures. We observe two effects: the 'bonus effect', where the introduction of a hint increases the likelihood of gene structures that are compatible with the hint relative to gene structures that are not compatible with the hint (we say the compatible gene structures are 'upvalued'); and the 'malus effect', where exons and signals that are not supported by hints become less likely than in the *ab initio *model. For example, suppose we have searched for extrinsic evidence about genes in a sequence region and have found no hints. Then the posterior probability of a gene in this region in the above model is smaller than its posterior probabilty in the *ab initio *model. Unsuccessful searches for hints tend to result in a prediction with fewer exons or genes. 'No information' is also information. Both effects are illustrated using an example in Figure 1.

#### Hints about introns

Suppose the input sequence *s *can be well aligned to an EST or a protein sequence, in such a way that a segment *s*[*a*, *b*] of *s *extending from position *a *to position *b *is aligned to a large gap in the other sequence. Furthermore, suppose that the splice site dinucleotide consensus occurs at the boundaries *a *and *b *of that segment. Then it is reasonable to assume that this segment is likely to be an intron. We would like to be able to formulate this as 'intron hint', which states that there is a likely intron extending exactly from *a *to *b*. The presence of such a hint should upvalue every gene structure that is compatible with the hint and has an intron going from *a *to *b*.

This problem turns out to be tricky. If HMM based gene prediction programs modeled complete introns as an emission from one state only, the time to compute the commonly used Viterbi recursion for intron states would be proportional to the maximum allowed intron length. As introns can be hundreds of kilobases long, for performance reasons programs do not model the intron as a complete emission but model introns piecewise using states that emit just one base at a time [7,9,14,15] or a bounded number of bases [2,16]. Therefore, the Viterbi algorithm does not allow a gene structure to be upvalued based on a complete long intron.

The Viterbi algorithm does allow, for example, the probability of the gene structures to be upvalued by a constant factor for each base of an intron that overlaps the interval from *a *to *b*. However, such a positionwise bonus would give a bonus to a predicted intron that just overlaps with [*a*,*b*] and has different splice sites and also would depend too strongly on the length of the intron hint [13,17].

Using hints to the splice sites is not solving the problem, either, because then gene structures that have splice sites at *a *and *b *but have an exon in this range are upvalued although they are not compatible with the hint and contradict the alignment. Of course, it is easily possible to force a program to predict an intron exactly from *a *to *b*, but this does not account for the fact that such hints can be wrong.

We present here a heuristic approach that allows hints about introns to be incorporated into a HMM. The idea is that the possibility of emitting a complete intron in one step exceptionally arises when the intron is exactly as given by a hint. This way, gene structures that exactly obey an intron hint can be upvalued arbitrarily and the overall additional computational cost is proportional only to the number of intron hints.

To illustrate the concept, just consider the forward strand of the DNA sequence and assume that the HMM has one state *acc *that models the first exonic base downstream of an acceptor splice site and one state *don *that models the first exonic base upstream of a donor splice site. Let *q *be a state of the GHMM and let *i *be a position in the sequence *s*. If *q *= *acc *or *i *= *b *+ 1 is not the exon base following the intron hint, then we use the normal Viterbi-recursion:

*γ*_*q,i *_= max_*q'*_t_*q',q*_*γ*_*q',i*-1_*p*(*s*_*i*_|*q*)

In the maximum *q' *ranges over all states, *t*_*q',q *_denotes the probability of the transition from state *q' *to state *q *and *p*(*s*_*i*_|*q*) the probability of the *i*th base of *s *under the model of state *q*. Without the adjustment below, the Viterbi-variables *γ*_*q,i *_have the usual meaning [18]. In those cases where *q *= *acc *and where *i *= *b *+ 1, we use a different recursion formula and take into account, as an additional alternative, that the intron is emitted in one step.



Here, *p*(*s*[a,b]|*intr*.) is the probability of the intron sequence of the hint under the intron model and includes the probability of the length. It is identical to the probability of the intron when modeled piecewise using a sequence of emissions and transitions. *r *is a bonus factor that upvalues the probability of gene structures that are compatible with the intron hint; *r *could depend on the reliability of the hint. In particular, this method can be used to enforce some introns; however, here we chose a fixed *r *= 10. The effect of the above method is that the probability of gene structures that are completely compatible with the hint are upvalued by a factor of *r *relative to all other gene structures.

### Predictions of AUGUSTUS on the ENCODE regions

In each of the categories of the EGASP workshop, we used the same program, AUGUSTUS, to predict the genes. The difference lies in the set of hints that are given to AUGUSTUS. In each case the hints were generated automatically using the available information in the respective category. The hints are given to AUGUSTUS in the form of a file in GFF format. In none of the categories was human intervention necessary. All predictions were made on the repeat masked sequence.

#### Ab initio single genome

The program AUGUSTUS has been described in [2]. We here only summarize it briefly and state what is additionally relevant when running the human version on large sequences. When run as an *ab initio *single genome gene finder, AUGUSTUS takes as input a DNA sequence *s *only and proceeds internally, that is, hidden from the user, as follows. It cuts *s *into non-overlapping pieces of length ≤200 kb, such that the cutting points are likely to be in the intergenic region. These cutting points are chosen using preliminary predictions of the model. For each such piece, the GC content is computed and a parameter set out of 10 possible GC content dependent sets is chosen. Then the most likely gene structure for each piece is searched using the Viterbi algorithm and the results are mapped back to the original sequence.

Currently, AUGUSTUS just predicts the coding sequence (CDS) and not the untranslated regions. In EGASP, AUGUSTUS predicted just one transcript per gene. However, after the workshop it has been extended to be able to predict multiple transcripts per gene [19]. The human version of AUGUSTUS was trained on a training set with 1,286 genes retrieved in 2002 from GenBank. This training set is available from the AUGUSTUS web server [20]. The running time for the 21.9 Mb of the EGASP test regions was 4 hours on a single processor PC of 2.4 Ghz. Everything stated above also applies to the following three sections when hints are used as additional input to AUGUSTUS; in particular, the hints do not slow down the program significantly.

#### Dual genome based

In this section we describe how we predicted the genes in a human input sequence *s *using the mouse genomic sequence as additional information. The method is based on the observation that functional regions tend to be more conserved between human and mouse than nonfunctional regions. Conversely, high conservation of a segment pair at the amino acid level is (weak) evidence that this segment is coding in both species.

The application flow of our method is as follows (see also [21]). First, we parse the precomputed UCSC BLASTZ [22] alignments of human with mouse to obtain large (up to 100 kb) alignable human/mouse sequence pairs. Second, for each such sequence pair we use CHAOS [23] to find alignment anchor points. Third, between the anchor points we use DIALIGN [24] to find fragments conserved on the peptide level. Fourth, we process the DIALIGN fragments and make a set of hints *h *from them. Fifth, AUGUSTUS predicts genes on *s *using the hints *h*.

The alignments we use in step 1 were downloaded from [25]. In step 2 we break the alignment problem down into alignment problems of smaller size by anchoring the alignment at pairs of positions that are outstandingly similar, so-called anchor points. As the running time of DIALIGN is superlinear, this reduces the running time of the subsequent runs of DIALIGN.

**Figure 2:**
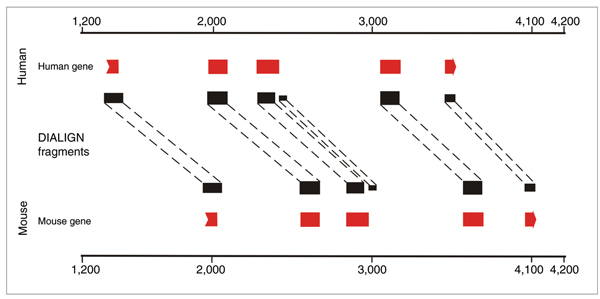
A syntenic human-mouse sequence pair and its DIALIGN alignment. Each sequence contains one gene with five exons (only CDS shown). The fragments are segment pairs with high similarity at the protein level.

DIALIGN (step 3) is an alignment program that we use in this context to find sequence pairs in human and mouse that have significantly high similarity at the amino acid level and are likely to correspond to coding exons. DIALIGN uses the term 'fragment' to denote a gap free local pairwise alignment. The weight of a fragment *f *when aligning two sequences of lengths *λ*_1_, *λ*_2 _is defined as:

*weight*(*f*) = -log*p*,

where *p *is the probability that two random sequences of lengths *λ*_1_,*λ*_2 _contain a fragment of the same length as *f *and with at least the BLOSUM62 score of *f*. DIALIGN then searches a chain of non-overlapping fragments, where the sum of the weights is maximal. The regions between the fragments remain unaligned. In the following step only the fragments of the optimal chain with a weight above the threshold of 20 were considered. Figure 2 shows an example of the DIALIGN alignment of a human-mouse sequence pair containing orthologous genes.

In particular, each such DIALIGN fragment defines a weight and in the human sequence an interval of sequence positions, a strand and a reading frame. In step 4, for each DIALIGN fragment several hints of type *exonpart *are generated for the human sequence. We now describe the hints that are generated for each single fragment. The interval of each of the hints is the interval of the fragment interval minus 33 base-pairs on each side. This cutoff accounts for the fact that, typically, some part of the introns flanking an exon are also conserved (Figure 2). We generate one hint for each reading frame and strand combination, that is, six hints in total per DIALIGN fragment. It is true that a DIALIGN hint specifies the strand and the reading frame but this information sometimes is wrong, although the fragment does indeed correspond to an exon (see the example in Figure 1). The reason for this is that an exon pair with very high sequence similarity will usually have high similarity at the amino acid level in any reading frame or on either strand. Nevertheless, for those fragments for which the hints interval indeed fell completely into a coding exon, the strand specified by DIALIGN was correct 72% of the time and the reading frame specified by DIALIGN was correct 61% of the time (estimated on a subset of the ENCODE training regions). Thus, the strand and reading frame given by DIALIGN contains useful information as it is much more often correct than guessing would be. However, it is not correct often enough for AUGUSTUS to be able to rely on it.

The bonus a gene structure gets when a coding region fully contains the hint interval depends only on the DIALIGN weight, whether the strand is as given by DIALIGN and whether the reading frame is as given by DIALIGN (D). For the weight we distinguish only two cases, a weight of at least 45 or a weight below 45. The set of grades contains 2 × 2 × 2 = 8 elements:

{weight ≥45, weight < 45}

× {strand as D, strand not as D}

× {frame as D, frame not as D}

The 8 parameters for the bonus as well as the one parameter for the malus have been computed using the 13 ENCODE training regions. The computation of the bonus of each grade is based on the count of the hints of that grade that is compatible with the annotated gene structure. The 8 relative bonuses range between 2.3 and 85.1, the latter for hints with weight ≥45 and both strand and score as given by Dialign. Therefore, the likelihood of a gene structure that has an exon that is supported by a DIALIGN fragment with weight greater than 45 on the same strand and with matching reading frame is upvalued by a factor of 85.1 relative to other gene structures. The computation of the malus is based on how often annotated exons are not supported by any hint. Here, the malus is 0.951, which means, in particular, that an exon of length *λ *that is not supported by any DIALIGN fragment is punished by the factor 0.951^*λ*^. For details on the parameter estimation see [3,13].

The running time of the first four steps of the automatic pipeline described above is dominated by the running time of DIALIGN. For the 31 test ENCODE regions encompassing 21.9 Mb, these steps took about 3 hours on a single CPU. An example of the constructed hints is shown in Figure 1.

#### EST and protein based

To make use of evidence derivable from ESTs and protein sequences, we automatically generated hints about the gene structure in the input sequence *s *using an EST and a protein database. For the EST database we used *est_human *from the NCBI. For the protein database we used the NCBI *nr *database. The application flow in this category of predictions is as follows. First, we search for local alignments of *s *to ESTs using WUBLASTN [26] (parameters -Q 15 -R 15 -B 250 -V 250) and to protein sequences using WUBLASTX (parameters -B 250 -V 250). Second, a program called AGRIPPA [27] parses the BLAST alignments of step 1, starts new WUBLASTX searches and generates a set of hints *h*. Third, AUGUSTUS predicts genes on *s *using the hints *h*.

The EST alignments are used to generate hints of types *exon, intron, exonpart, dss *and *ass*. The protein alignments are used to generate hints of all seven types. In addition, AGRIPPA generates hints using a combined EST and protein search in the following way. After the EST database has been used to partially reconstruct the mRNA, each presumable part *τ *of an mRNA sequence is searched against the protein database. The idea behind this is that parts of *τ *that are aligned to an amino acid sequence are relatively likely to be coding. Thus, this search is a means of separating non-coding exons from coding exons. For details on the generation of hints from transcribed data see [3,13].

The reliability of the hints depends on whether they were derived from ESTs, proteins or from a combined search. For example, hints to donor splice sites were much more reliable when they came from protein alignments than when they were from EST alignments. When an identical hint is derived both by EST and by protein alignment, we keep only the hint from the more reliable source. We introduce three grades for hints according to their source of information. One grade is assigned to all hints from ESTs, one grade is assigned to all hints from proteins and one grade is assigned to hints from a combined EST-protein search. None of the hints depend on the BLAST e-value. We treated each entry in the protein database equally, no matter if the species was human or not. Also, we treat each entry in the EST database equally. We again estimated the parameters for the hints on the 13 training regions. For details we refer to [3,13].

In the above pipeline, by far the most time consuming step is the blast runs, particularly the WUBLASTX run from step 1 against the protein database, which requires many computing resources. Blasting the 31 test sequences against the *nr *database took about 50 CPU days. However, when the BLAST results have been precomputed and the BLAST output is given to AGRIPPA as input it takes time in the order of minutes to generate the hints.

#### EST, protein and dual genome based

The evidence about the gene structure coming from genome to genome comparisons extends and partially complements the evidence from the similarity to transcribed sequences. In order to incorporate both kinds of information we simply take as a set of hints *h *the union of the two sets of hints described above, that is, we concatenate the GFF file containing the hints from ESTs and proteins and the GFF file containing the hints from comparisons to the mouse genome. The set of possible grades for each type of hint also encompasses the union of the two sets of possible grades of the above two categories. For example, for hints of type *exonpart *there are now 8 + 1 + 1 + 1 = 11 grades possible in order to distinguish between the reliabilities of the 8 grades of hints from DIALIGN and of hints from protein alignments, from EST alignments and from combined EST-protein searches. The parameters for this new configuration have again been estimated on the 13 training regions.
